# Supplementary material for: The Effect of RBP4 on microRNA Expression Profiles in Porcine Granulosa Cells
Source: Animals (Basel). 2021 May 13;11(5):1391. doi: 10.3390/ani11051391 (PMC8153112; doi:10.3390/ani11051391)
Supplement: Supplementary file 1 [file animals-11-01391-s001.zip › animals-1159236-supplementary/supplement/Table S1.pdf]

Table S1 List of primers used for RT-qPCR, reverse transcription

Primer name	Sequence 5'-3'
RBP4-F	GGGCGTAGCATCCTTCCT
RBP4-R	TCCGATTTGCCATCACAG
KIT-F	GAACAAATCCATGCCCACAC
KIT-R	CTCGACAACCTTCCACTGTAC
SEMA6D-F	CGAAGGTGGACTATGTTCAGG
SEMA6D-R	TCAGGTTTTAAGGACGGTGTC
RAB3B-F	AATCTTTTCTCCCAGCCCTG
RAB3B-R	CCCTACTTCCCCACTGATTTC
GAPDH-F	TGGGCATGAACCATGAGAAG
GAPDH-R	CCACGATGCCGAAGTTGTC
miR-194a-5p-F	CGCGTGTAACAGCAACTCCA
miR-194a-5p-R	GTGCAGGGTCCGAGGT
miR-132-F	GCGCGTAACAGTCTACAGCCA
miR-132-R	GTGCAGGGTCCGAGGT
miR-98-F	CGCGCGTGAGGTAGTAAGTTGT
miR-98-R	GTGCAGGGTCCGAGGT
miR-1343-F	CTCCTGGGGCCCGCACCTCT
miR-1343-R	GTGCAGGGTCCGAGGT
miR-145-5p-F	CGGTCCAGTTTTCCCAGGA
miR-145-5p-R	GTGCAGGGTCCGAGGT
miR-148a-5p-F	GCGCGAAAGTTCTGAGACACT
miR-148a-5p-R	GTGCAGGGTCCGAGGT
miR-32-F	CGCGCGTATTGCACATTACTAA
miR-32-R	GTGCAGGGTCCGAGGT
miR-135-F	CGCGTATGGCTTTTCATTCCT
miR-135-R	GTGCAGGGTCCGAGGT
PC-5p-93122-F	CGCGCGTTAGTTGGTGGAG
PC-5p-93122-R	AGTGCAGGGTCCGAGGTATT
PC-3p-102262-F	GCGCGCCTATTGGGAAAG
PC-3p-102262-R	AGTGCAGGGTCCGAGGTATT
miR-769-5p-F	CGCGTGAGACCTCTGGGTTC
miR-769-5p-R	AGTGCAGGGTCCGAGGTATT
miR-7142-3p-F	GCGTTTGTTGGCTCCTCTG
miR-7142-3p-R	AGTGCAGGGTCCGAGGTATT
miR-7857-3p-F	GCGATTGTTCTCCAACCTGG
miR-7857-3p-R	AGTGCAGGGTCCGAGGTATT
miR-6782-3p-F	CGTGACCTCTGGTCTCCCG

miR-6782-3p-R	AGTGCAGGGTCCGAGGTATT
miR-1277-F	CGCGCGCGTATATATATATATGTA
miR-1277-R	AGTGCAGGGTCCGAGGTATT
PC-5p-17154-F	CGCGCGAAGTGTTGTGTTC
PC-5p-17154-R	AGTGCAGGGTCCGAGGTATT
miR-193a-5p-F	TGGGTCTTTGCGGGCG
miR-193a-5p-R	AGTGCAGGGTCCGAGGTATT
U6-F	CTCGCTTCGGCAGCACA
U6-R	AACGCTTCACGAATTTGCGT
Reverse transcription primers	
miR-194a-5p	GTCGTATCCAGTGCAGGGTCCGAGGTATTCGCACTGGATACGACTCCACA
miR-132	GTCGTATCCAGTGCAGGGTCCGAGGTATTCGCACTGGATACGACCGACCA
miR-98	GTCGTATCCAGTGCAGGGTCCGAGGTATTCGCACTGGATACGACAACAAT
miR-1343	GTCGTATCCAGTGCAGGGTCCGAGGTATTCGCACTGGATACGACGCGAGA
miR-145-5p	GTCGTATCCAGTGCAGGGTCCGAGGTATTCGCACTGGATACGACAGGGAT
miR-148a-5p	GTCGTATCCAGTGCAGGGTCCGAGGTATTCGCACTGGATACGACAGTCGG
miR-32	GTCGTATCCAGTGCAGGGTCCGAGGTATTCGCACTGGATACGACTGCAAC
miR-135	GTCGTATCCAGTGCAGGGTCCGAGGTATTCGCACTGGATACGACTCACAT
PC-5p-93122	GTCGTATCCAGTGCAGGGTCCGAGGTATTCGCACTGGATACGACGAATCG
PC-3p-102262	GTCGTATCCAGTGCAGGGTCCGAGGTATTCGCACTGGATACGACGGAAAC
miR-769-5p	GTCGTATCCAGTGCAGGGTCCGAGGTATTCGCACTGGATACGACAGCTCA
miR-7142-3p	GTCGTATCCAGTGCAGGGTCCGAGGTATTCGCACTGGATACGACACACTT
miR-7857-3p	GTCGTATCCAGTGCAGGGTCCGAGGTATTCGCACTGGATACGACAAAGAG
miR-6782-3p	GTCGTATCCAGTGCAGGGTCCGAGGTATTCGCACTGGATACGACACAGGG
miR-1277	GTCGTATCCAGTGCAGGGTCCGAGGTATTCGCACTGGATACGACCATACG
PC-5p-17154	GTCGTATCCAGTGCAGGGTCCGAGGTATTCGCACTGGATACGACGCCACT
miR-193a-5p	GTCGTATCCAGTGCAGGGTCCGAGGTATTCGCACTGGATACGACT

	CATCT
