# Supplementary figures and images for: The Effect of RBP4 on microRNA Expression Profiles in Porcine Granulosa Cells
Source: Animals (Basel). 2021 May 13;11(5):1391. doi: 10.3390/ani11051391 (PMC8153112; doi:10.3390/ani11051391)

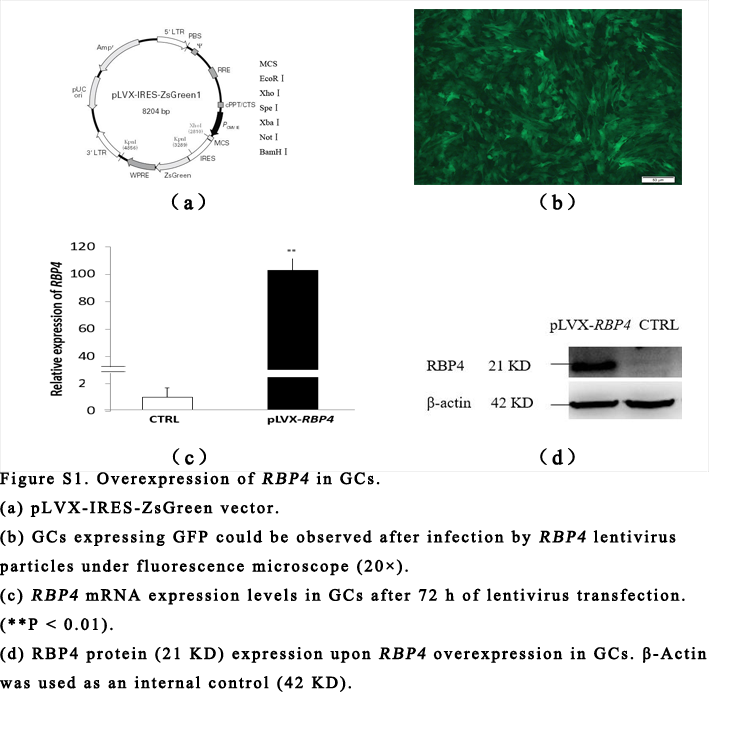

Supplement: Supplementary file 1 [file animals-11-01391-s001.zip › animals-1159236-supplementary/supplement/Figure S1.tif]
